# Real-World Effectiveness of the Peer-Led Honest, Open, Proud Programme for Self-Stigma Among Adults With Mental Illness: A Pragmatic, Multicentre, Randomised Controlled Trial

**DOI:** 10.1016/j.lanepe.2026.101751

**Published:** 2026-06-19

**Authors:** Nicolas Rüsch, Claudia Schulz, Chiara Weisshap, Carolin Knoke, Julia Wöhrle, Kathrin Reichmann, Ronja Büchner, Christian Sander, Georg Schomerus, Irina Papazova, Naiiri Khorikian-Ghazari, Wolfgang Strube, Patrick W. Corrigan, Reinhold Kilian, Christian Ruckes, Alkomiet Hasan, Peter Brieger, Nathalie Oexle

**Affiliations:** a Department of Psychiatry II, Ulm University and BKH Günzburg, Germany; b German Centre for Mental Health (DZPG), partner site Mannheim-Heidelberg-Ulm/ZIHUb, Germany; c Department of Psychiatry and Psychotherapy, Medical Faculty, Leipzig University, Germany; d Department of Psychiatry, Psychotherapy and Psychosomatics, Faculty of Medicine, University of Augsburg, Germany; e German Centre for Mental Health (DZPG), partner site München/Augsburg, Germany; f Illinois Institute of Technology, Chicago, USA; g Interdisciplinary Centre for Clinical Trials, Mainz, Germany; h kbo-Isar-Amper-Klinikum, Region of Munich, Germany

**Keywords:** Randomised-controlled trial, Mental illness, Honest, Open, Proud, Coming Out Proud, Disclosure, Stigma, Self-stigma, Secrecy, Peer support

## Abstract

**Background:**

The stigma of mental illness is a key barrier to recovery, help-seeking and social inclusion of people with mental disorders. Faced with stigma and discrimination, many struggle with the decision whether to disclose their condition to others. Honest, Open, Proud (HOP) is a peer-led group programme that supports participants with disclosure decisions. This study examined HOP’s real-world effectiveness to reduce self-stigma and to improve secondary clinical and social outcomes as well as its cost-effectiveness.

**Methods:**

In this open-label, pragmatic parallel 2:1-randomised trial, we included working-age adults with mental illness across nine sites in Germany, recruited in clinical and community settings. Participants were randomly assigned to HOP and treatment as usual (TAU, 2/3), or to TAU alone (1/3). Outcomes were assessed by self-report pre (baseline), post (6 weeks) and at 6-month follow-up. The primary endpoint was self-stigma at 6 weeks. Intervention effects were analysed by intention-to-treat analysis. The trial was pre-registered on DRKS (https://drks.de/search/en/trial/DRKS00033314).

**Findings:**

From February to November 2024, 457 participants (mean age 42 years, 66% female, 92% German born) were included in the trial; 306 were randomised to HOP and TAU, and 151 to TAU alone. Compared to TAU, HOP participants showed significantly less self-stigma at week 6 (−1.83, 95%-CI −2.96 to −0.70, d = −0.24, p = 0.0015) and at 6-month follow-up (−1.20, 95%-CI −2.36 to −0.05, d = −0.16, p = 0.042). In terms of secondary outcomes, HOP had significant positive effects on stigma stress, depressive symptoms, help-seeking intentions, quality of life, recovery, shame, and social inclusion at 6 weeks. In an economic evaluation HOP was cost-effective in terms of quality of life gains. There were no project-related adverse events.

**Interpretation:**

HOP effectively reduces self-stigma among adults under real-world conditions and should be offered in addition to usual care in clinical and community settings.

**Funding:**

Federal Department of Health, Germany (2523FSB22A, 2523FSB22B).

**Research in context**

**Evidence before this study**

In addition to symptoms and disabilities caused by mental disorders, public stigma (“People with mental illness are bad”) and internalised or self-stigma (“Because I have a mental illness, I must be bad”) are an enormous burden on people with mental illness, carers and society, as evidenced by the 2022 *Lancet* Commission on ending stigma and discrimination in mental health. While there is strong evidence that public stigma is effectively reduced by social contact between people with and without mental illness, the evidence on programmes to reduce self-stigma is less clear. Existing programmes to reduce self-stigma use psychoeducation, such as Ending Self-Stigma, or narrative-cognitive approaches, such as Narrative Enhancement and Cognitive Therapy. ‘Honest, Open, Proud’ (HOP), on the other hand, was developed as a peer-led group programme that supports participants with mental illness in their decisions whether to disclose their condition to others, with the aim to reduce self-stigma. As mental illness is usually a concealable stigmatised trait, disclosure decisions are a key reaction in dealing with public and self-stigma. HOP empowers participants to make strategic choices whether or not to disclose, depending on their personal situation and the environment. Increased empowerment is related to decreased self-stigma, so this approach is meant to reduce self-stigma. Prior to this study, we conducted a literature search on HOP as a peer-led programme with the search terms HOP and related terms (e.g., HOP’s previous name ‘Coming Out Proud’), and with ‘self-stigma’ or ‘internalised stigma’ and related terms, without language restrictions and from database inception until 1 October 2020 in PubMed and PsycInfo, updated on 31 July 2025. In our first search, we found four RCTs of HOP and published a meta-analysis, including a fifth RCT yet unpublished at the time, that showed small significant effects of HOP on self-stigma at short-term follow-up compared to treatment as usual (standardised mean difference −0.24). In our search update, we found four new HOP trials, two Chinese trials for adults with severe mental illness, one German trial for soldiers with mental illness, and one feasibility trial for young people with psychosis in the UK. In summary we found a limited evidence base: Trials were small, had short-term follow-up usually six weeks after baseline, most had evaluated older HOP versions that unlike the current HOP programme did not include a booster session, and the previous trials had not been conducted as pragmatic multi-site trials across different settings and urban as well as rural regions or had not included people with different conditions. There was also very limited data on HOP’s effects on social outcomes such as employment and on HOP’s cost-effectiveness.

**Added value of this study**

To the best of our knowledge, this is the largest HOP trial conducted so far. In diverse settings, among participants with different mental disorders, across urban and rural regions, and with broad inclusion criteria, HOP effectively reduced self-stigma and had positive effects on depressive symptoms, stigma-related stress, shame, recovery, help-seeking intentions, social inclusion, and quality of life. Some effects, incl. on self-stigma and depressive symptoms, remained significant at five-month follow-up. There was a positive, non-significant trend of HOP’s effect on re-employment in a subsample of participants who were unemployed at baseline. In a health economic evaluation HOP provided good value for money in terms of quality of life gains.

**Implications of all the available evidence**

In real-life settings, HOP is an effective and cost-efficient intervention to reduce self-stigma as well as to improve clinical and social outcomes among adults with mental illness in addition to usual care. Therefore we recommend that HOP should be offered to adults with mental illness as a transdiagnostic, peer-led group programme to reduce the impact of mental illness stigma and to improve broader social and clinical outcomes. HOP is non-commercial, brief and flexible and can be implemented across different settings.

## Introduction

Adults with mental illness often experience stigma and discrimination which can have a severe impact on long-term clinical, social and vocational functioning.^1^^,^^2^ Many people with mental illness struggle with the decision whether to disclose their condition to others or not.^3^^,^^4^ Disclosure may lead to labeling and discrimination, on the other hand it offers opportunities for social support, facilitates help-seeking, increases authenticity and decreases the stress associated with secrecy.^5^ Given the consequences of disclosure, this can be a challenging decision and individuals may weigh the pros and cons differently.

Therefore Honest, Open, Proud (HOP) was developed by Patrick W. Corrigan and colleagues in Chicago, USA, as a peer-led group programme that supports participants with mental illness in their disclosure decisions (www.hopprogram.org) with the aim to reduce self-stigma.^5^ A first meta-analysis of our group, based on five small randomised-controlled trials (RCTs),^6^ supported HOP’s efficacy to reduce stigma stress, or the perception of individuals with mental illness that stigma-related harm exceeds their coping resources, and self-stigma (i.e., the internalization of negative stereotypes: “I must be bad, because I have a mental illness”). However, these trials were small with a combined total of HOP participants across all five trials below 200; there was no follow-up beyond one month after HOP, and the HOP versions in these RCTs mostly did not use a booster session. So far, HOP for adults has only been evaluated in one European RCT,^7^ other European trials investigated HOP for adolescents^8^ or HOP for soldiers with mental illness.^9^ To the best of our knowledge, no version of HOP has been evaluated anywhere in a pragmatic trial^10^ across different settings under usual-care conditions and with a follow-up beyond three months.

The aim of this study was therefore to examine HOP’s real-world effectiveness to reduce self-stigma and to improve secondary outcomes among adults with mental illness in a pragmatic multi-site RCT. We also investigated HOP’s effectiveness five months after the programme, its cost-effectiveness in relation to quality of life gains, and HOP’s effects on re-employment.

Our primary hypothesis was that, compared to control group participants, HOP participants report lower self-stigma after the end of the HOP programme (six weeks after baseline, post/T1). Our second hypothesis was that, compared to the control group at T1, HOP participants have lower scores in stigma stress, depressive symptoms, and shame; and have higher scores in quality of life, recovery, social inclusion and more positive attitudes to help-seeking and to disclosure. Our third hypothesis was that, compared to the control group at follow-up (six months after baseline/T2), HOP participants have lower scores in self-stigma, stigma stress, depressive symptoms, and shame; and have higher scores in quality of life, recovery, social inclusion and more positive attitudes to help-seeking and to disclosure.

Our fourth hypothesis was that among trial participants who are unemployed at baseline, HOP leads to higher re-employment rates at T2. Our fifth hypothesis was that HOP is cost-effective with regard to quality of life gains.

## Methods

### Trial design and participants

In this two-arm open-label parallel pragmatic 2:1-RCT, participants were randomly assigned to HOP, combined with treatment as usual (TAU), or to a control group that received only TAU. We chose a 2:1-randomisation because in previous HOP trials of our group a 1:1-randomisation had made it difficult to recruit participants to the trial and to start new HOP groups.^7^, ^8^, ^9^ Participants were recruited from nine sites in Germany (Augsburg, Günzburg, Heidelberg, Konstanz, Leipzig, Munich, Regensburg, Stuttgart, Ulm) from 13 February to 25 November 2024. At all sites, participants were recruited in different settings: inpatient, day clinic and outpatient psychiatric or psychotherapeutic facilities; peer-support, mutual support groups, recovery colleges; via articles about the project in print and online media, usually in local newspapers at the different sites; the German website on HOP (iwsprogramm.org); primary care services; counselling centres in the community; and psychological counselling services for students.

Inclusion criteria for all participants were broad and assessed by project staff at each site, based on the self-reported information provided by potential participants during the screening: at least one self-reported current axis-I or axis-II disorder according to ICD-10 in response to a list of major diagnostic categories; age 18 to 60 since we wanted to assess potential effects of HOP on employment; ability to provide written informed consent; and sufficient German language skills. Exclusion criteria were intellectual disability; organic disorder; or diagnosis of only a substance- or alcohol-related disorder, without non-substance related current psychiatric comorbidity, since disclosure of these disorders is not a topic specifically discussed in this HOP programme and the stigma of substance use disorder differs from mental illness stigma.^11^

The potential occurrence of project-related adverse events, such as psychological distress caused by HOP groups or by assessments, was not assessed with a standardised instrument, but was carefully discussed every two weeks in calls between the authors, HOP group facilitators as well as project teams and research assistants at all sites.

HOP is a peer-led programme, therefore this study was designed, conducted, analysed and written up with the active participation of people with lived experience of mental illness (CSch, JW as co-authors and others) who advised on the choice of the primary endpoint, recruitment, training of group facilitators, quality of programme delivery during the trial (e.g., in terms of programme fidelity), assessment, analyses, interpretation, and dissemination of results. HOP group facilitators were trained by a peer (CSch or JW) and a researcher (NR).

### Sample size

A recent meta-analysis suggested small-to-moderate effects of HOP on self-stigma.^6^ We therefore based our sample size estimate on d = 0.4, a power of 80%, two-sided alpha of 0.05, and a 12% adjustment for the 2:1 randomisation,^12^ resulting in N = 224. During this trial, we experienced high demand for trial participation. Without increasing the recruitment period or funding, with approval of all ethics committees, and blinded to outcomes, we decided to increase the sample size in order to increase the power to detect intervention effects and to improve the precision of our estimates. Based on a power of 90% to detect an HOP effect on self-stigma at T1, alpha of 0.05, 388 participants were sufficient to detect a small-to-medium effect (d = 0.35) on self-stigma, allowing for a dropout rate of 15%, resulting in 456 participants. That said, the sample size estimate did not account for the partially nested design, reducing power.

### Randomisation

After completing the baseline assessment (T0), participants were 2:1-randomly assigned to the intervention (HOP + TAU) or to the control group (TAU alone) by block randomisation separately for each study centre; 2/3 were randomised to HOP, 1/3 to TAU alone. Randomisation lists and closed opaque envelopes were generated by the Institute of Epidemiology and Medical Biometry, University of Ulm, Germany. Block sizes were 3, 6 or 9 in random order. Once a participant had completed the baseline (T0) assessment, research staff at each site opened a new envelope for this participant. Blinding of participants was not feasible, and research staff as well as statisticians were not blinded, as all outcomes were assessed by self-report. To reduce the risk of contamination between trial arms, HOP participants were asked not to share HOP materials with control group participants.

### Intervention

HOP’s goal is to support participants with the decision whether to disclose their mental illness in different settings. HOP was developed by Patrick W. Corrigan and colleagues in the US.^5^ It was previously known as ‘Coming Out Proud’ and is now called ‘Honest, Open, Proud’ (www.hopprogram.org). For a previous trial,^7^ we had translated and adapted HOP for the German speaking context which involved participatory work with service users; in German HOP is known as IWS (‘In Würde zu sich stehen’, translatable as: To stand up for yourself in dignity; www.iwsprogramm.org).

As disclosure decisions depend on the setting and are very individual, HOP discusses levels of disclosure, settings of disclosure and how to choose persons to disclose to. It is not HOP’s aim to make participants disclose, but to empower them to make their own decision. HOP is a peer-led group programme that covers three main themes in three 2-h lessons; (i) pros and cons of disclosure, (ii) levels of disclosure and choosing persons to disclose to, if one wants to disclose; and (iii) ways to tell one’s story. A fourth booster lesson, three weeks after lesson 3, reflects on participants’ experiences with their (non-)disclosure since lesson 3. All lessons are structured by vignettes, first-person accounts, worksheets, tables and role plays in the HOP workbook.

HOP was conducted by two peers per group in four 2-h sessions with sessions in weeks 1, 2, 3 and 6. Prior to the RCT, all peer group facilitators were trained in HOP and ran at least one HOP practice group (practice groups did not include trial participants). Manual fidelity in the practice groups needed to be >80% before the respective group facilitators could run groups with trial participants. The group size during the trial varied from 2 to 11 participants (M = 5.75, SD = 1.69, median 5).

TAU consisted of any mental health service or other support that participants chose, whatever that might be. About 3 in 4 participants were in outpatient mental health care at baseline, about 30% were treated by a general practitioner, and 1 in 5 participated in a mutual support group (Table 1).

**Table 1:** Clinical characteristics of trial participants at baseline, including self-reported diagnoses and service use (participants could list more than one for both).

	All participants (N = 457) M (SD), or n (%)	HOP (n = 306) M (SD), or n (%)	Control (n = 151) M (SD), or n (%)
Depressive disorder	326 (71%)	224 (73%)	102 (68%)
Bipolar disorder	60 (13%)	44 (14%)	16 (11%)
Anxiety disorder	159 (35%)	109 (36%)	50 (33%)
Posttraumatic stress disorder	139 (30%)	95 (31%)	44 (29%)
Psychosis or schizophrenia	93 (20%)	59 (19%)	34 (23%)
Alcohol or substance use disorder	38 (8%)	23 (8%)	15 (10%)
Obsessive-compulsive disorder	44 (10%)	31 (10%)	13 (9%)
Borderline personality disorder	55 (12%)	38 (12%)	17 (11%)
Eating disorder	62 (14%)	43 (14%)	19 (13%)
ADHD	64 (14%)	45 (15%)	19 (13%)
Autism	20 (4%)	16 (5%)	4 (3%)
Years since first psychiatric diagnosis	14.2 (10.3), range 0–52	13.8 (10.0), range 0–40	15.1 (11.0), range 0–52
Number of psych. inpatient treatments	3.9 (4.4), range 0–26	3.7 (4.2), range 0–26	4.3 (5.0), range 0–25
Number of compulsory psychiatric admissions	0.4 (1.3), range 0–16	0.4 (1.2), range 0–16	0.5 (1.5), range 0–10
Current service use for mental illness			
Inpatient/day clinic mental health care	64 (14%)	48 (16%)	16 (11%)
Outpatient mental health care (psychiatrist or psychotherapist)	342 (75%)	228 (75%)	114 (76%)
General practitioner	131 (29%)	88 (29%)	43 (29%)
Mutual support group	85 (19%)	55 (18%)	30 (20%)
Psychosocial counselling service	77 (17%)	55 (18%)	22 (15%)
No use of any support or service	27 (6%)	18 (6%)	9 (6%)

### Fidelity

In order to check manual fidelity, a checklist covering the HOP workbook content was adapted from a previously used HOP fidelity scale.^7^ In one out of four sessions, a research assistant was present in the background of the group session and completed the fidelity checklist. Fidelity was high with, on average, 98% for lesson one, 97% for lesson two, 96% for lesson three, and 98% for lesson four. Mean fidelity across lessons and sites was 97%.

### Measures

All outcomes were measured by self-report at three times: at baseline before randomisation (Pre/T0); 6 weeks after baseline, i.e., after the booster session for HOP participants (Post/T1); and 6 months after baseline (Follow-up/T2). We assessed self-stigma at T1 as primary endpoint, measured by the 5-item apply subscale of the Self-Stigma of Mental Illness Scale-Short Form (SSMIS-SF^13^; alphas in this study were 0.67/0.71/0.73 for T0/T1/T2, respectively), higher sum scores from 5 to 45 equaling more self-stigma. Self-stigma is one of the key barriers to recovery for people with mental illness,^14^ and the SSMIS-SF is one of the most common measures of self-stigma.

Secondary outcomes were stigma stress, assessed by the 8-item Stigma Stress Scale^15^; four items measured the primary appraisal of stigma as harmful (alphas in our study 0.91/0.92/0.94 for T0/T1/T2) and four items the secondary appraisal of perceived resources to cope with stigma-related harm (alphas 0.82/0.86/0.86). All eight items were rated from 1 to 7, higher mean scores indicating more harm or more coping resources, respectively. A stigma stress score was computed by subtracting perceived resources from perceived harm, with higher difference scores (range −6 to +6) indicating more stigma stress. Quality of life was measured by the 8-item EUROHIS-QOL,^16^ with higher sum scores from 8 to 40 indicating better quality of life (alphas 0.82/0.80/0.81). For the calculation of Quality Adjusted Life Years (QALYs) we used the EuroQol EQ-5D-3L^17^ combined with the German utility value set. We estimated QALY by means of the area under the curve (AUC) method.^18^ We extrapolated 6-month QALY to annual QALY by weighting utility estimators at T0, T1 and T2 equally in the AUC formula. Depressive symptoms were assessed with the 9-item Patient Health Questionnaire (PHQ-9^19^), higher sum scores from 0 to 27 equaling more depression (alphas 0.84/0.83/0.83). Social inclusion was rated by the 10-item Experiences of Social Inclusion Scale (ESIS^20^), with higher sum scores from 10 to 50 indicating higher social inclusion (alphas 0.84/0.85/0.86). Social Outcomes were rated by the 4-item Social Outcomes Index (SIX^21^), with greater sum scores from 0 to 6 indicating better social outcomes. Recovery was assessed with the 4-item Self-Identified Stage of Recovery (SISR-B^22^), higher sum scores from 4 to 24 indicating better recovery (alphas 0.74/0.75/0.72). Intention to seek professional help for mental health problems was measured with three items of the General Help-Seeking Questionnaire (GHSQ^22^^,^^23^), from 1/extremely unlikely to 7/extremely likely and yielding mean scores from 1 to 7 (alphas 0.55/0.57/0.59). Attitudes towards the disclosure of one’s mental illness among family/friends (7 items) or in work/education settings (7 items) were assessed by the Attitudes to Disclosure Questionnaire (AtDQ^24^), with higher mean scores from 1 to 7 indicating more positive attitudes towards disclosure for family/friends (alphas 0.83/0.86/0.87) and in work/education settings (alphas 0.84/0.85/0.85). This questionnaire can only be completed by participants who have social contact with the respective social domain, leading to expected higher rates of missing data (see Results below). Shame of one’s mental illness was measured by one item (“I am ashamed to have a mental illness”, from 1/not at all to 7/very much), and self-labeling with one item (from 1/“I am perfectly mentally healthy” to 7/“I am severely mentally ill”^25^).

### Statistical analyses

The analysis script is publicly available on the same platform as the pre-registration (https://drks.de/search/en/trial/DRKS00033314). Analyses were conducted in R (R Core Team 2023, version 4.4.2) and packages lme4,^26^ emmeans,^27^ and ggplot2.^28^ Descriptive sample characteristics were analysed using SPSS, version 29. Baseline characteristics of dropouts versus completers at T1 were compared using t-tests or chi-square tests. Intention-to-treat (ITT) analyses were conducted using linear mixed effects models that permit analysis of changes in repeated measures in the presence of missing data (MMRM).^29^^,^^30^ The model included time and group and their interaction as fixed effects, study site and baseline outcome scores as covariates, and a random intercept for participants. Group differences were calculated based on estimated marginal means. For p-values and 95%-confidence intervals Kenward-Roger degrees of freedom were used. Effect size *d* was calculated by dividing MMRM-estimated group differences by the pooled standard deviation at baseline. A sensitivity analysis of HOP effects on the primary endpoint (self-stigma at T1) accounted for the partially nested design, due to about 5–6 HOP participants receiving the group programme together, and separate groups were considered as fixed effect in MMRM. For ANCOVAs as sensitivity analysis, we used all available data; intervention effects were tested using group as between- and time as within-subject factors, and the baseline value of the respective outcome and site as covariates.

The longitudinal mediation model of the intervention effect on quality of life at follow-up/T2 (difference score T2-T0), mediated by stigma stress post intervention/T1 (difference score T1-T0), was tested with a structural equation model (SEM), using lavaan in R.^31^ Full Information Maximum Likelihood estimation accounted for missing data (n = 4). Confidence intervals for the parameter estimates were bootstrapped (number of replications = 1000). To assess the dose-response relationship, we calculated Spearman-rank correlation coefficients between the number of attended HOP sessions and change scores between T0 and T1 (or T2) for self-stigma, stigma stress, depressive symptoms and quality of life. For a cost-effectiveness analysis, we estimated programme costs per participant as numerator and the annual QALY difference as denominator. We imputed missing EQ5D values by the last observation carried forward (LOCF) method. We estimated the incremental cost-utility ratio variance by means of nonparametric bootstrapping with 10,000 replications and computed the cost effectiveness acceptancy curve at maximum willingness to pay (MWTP) threshold range between 0 and 125,000 €.^18^ For interpreting acceptancy rates, we refer to threshold values recently suggested for the German healthcare system.^32^

### Ethics approval

The trial was approved by the ethics committees of Ulm University (Nr. 137/23), Leipzig University (Nr. 347/23), Munich University (Nr. 23-0984), Heidelberg University (Nr. S-552/2023) and of the Medical Association of Baden-Württemberg (Nr. B-F-2023-081). All participants provided written informed consent after being fully informed about study procedures. Before including the first participant, the trial was registered in the German Clinical Trials Register (DRKS; https://drks.de/search/en/trial/DRKS00033314).

### Role of the funding source

The sponsor, the German Federal Department of Health, had no role in the study design; collection, analysis, and interpretation of data; in the writing of the report; and the decision to submit the paper for publication.

## Results

### Recruitment, baseline characteristics and dropout rates

**Fig. 1:**
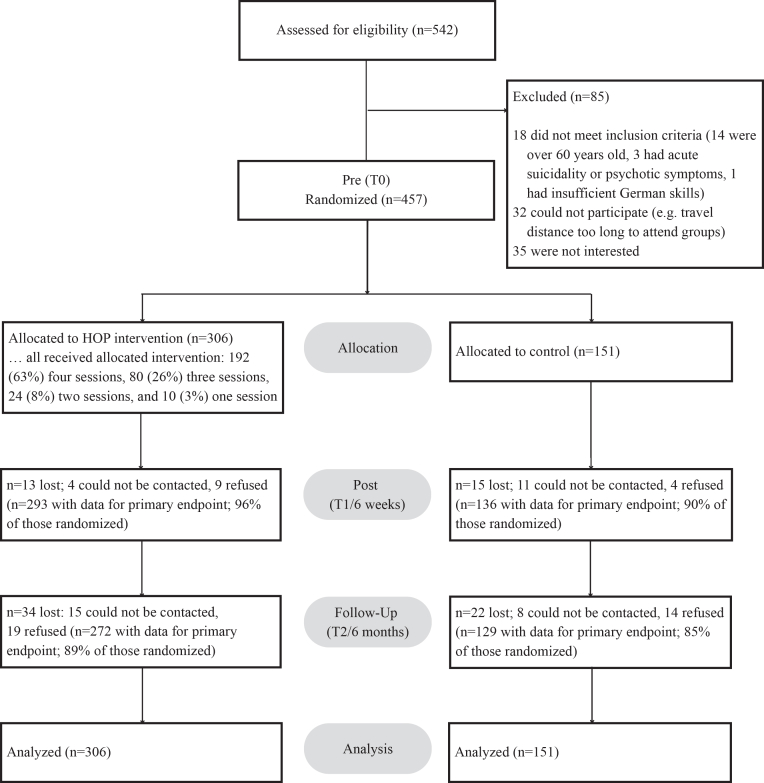
Flowchart of participants.

Across all sites and from 13 February to 25 November 2024, we included 72 participants from psychiatric or psychosomatic inpatient or day clinic settings, 38 from outpatient mental health services, 104 had heard about the project from flyers or social media, 33 from mutual help groups, 32 from psychosocial counselling services, 45 mouth-to-mouth from friends, 30 from print or online newspaper articles, 27 from day care centres, and one from a general practitioner. At each of the nine sites, between 20 (Konstanz) and 103 (Augsburg) participants were included in the study (M = 50.8, SD = 25.6). In total, 457 participants were included and randomised after giving written informed consent (Fig. 1).

Two thirds of all participants were female, on average in their early 40s and with 16 years of education. The vast majority was born in Germany, nearly half lived alone and about a quarter each was on disability benefits or unemployed (Table 2). In terms of clinical characteristics, the most commonly self-reported diagnosis was a depressive disorder, followed by anxiety disorder, PTSD and psychosis (all ≥ 20%, Table 1); on average, participants reported about two psychiatric diagnoses (M = 2.4, SD = 1.3, median 2). Participants had received their first psychiatric diagnosis about 14 years ago on average and had been in inpatient treatment four times; 84 (19%) had experienced at least one compulsory psychiatric admission. Three quarters currently received outpatient mental health care (Table 1).

**Table 2:** Socio-demographic baseline characteristics of trial participants.

	All participants (N = 457) M (SD), or n (%)	HOP (n = 306) M (SD), or n (%)	Control (n = 151) M (SD), or n (%)
Age, years	42.4 (12.0)	42.1 (12.2)	43.0 (11.5)
Female	301 (66%)	205 (67%)	96 (64%)
Years of education	16.1 (3.9)	16.0 (4.0)	16.3 (3.8)
Born in Germany	421 (92%)	283 (92%)	138 (91%)
Living alone	197 (43%)	136 (44%)	61 (40%)
Married or in long-term relationship (>2 years)	163 (36%)	105 (34%)	53 (35%)
Current disability benefits because of mental illness	124 (27%)	80 (26%)	44 (29%)
Currently unemployed	106 (23%)	69 (23%)	37 (25%)
Social Outcomes Index^a^	4.1 (1.3)	4.1 (1.3)	4.1 (1.3)

a Social Outcomes Index (SIX^21^), 4 items, higher sum scores from 0 to 6 indicating better social outcomes in terms of work, living, family status and social contacts.

All participants completed questionnaires at baseline (T0), 429 (94%) completed the post assessment (T1/6 weeks after baseline, for HOP participants after the booster session), and 401 (88%) the follow-up assessment (T2, six months after baseline and 5 months after HOP) (Fig. 1). These are the numbers of participants included in the analyses, if not stated otherwise in the Results (for some variables, there was a maximum of three additional missing data). Baseline characteristics of the 28 participants lost at T1 did not differ significantly from the 429 completers (Table 3). About two thirds of HOP participants attended all four sessions (Fig. 1). No project-related adverse events occurred among trial participants.

**Table 3:** Baseline characteristics and group assignment of completers versus dropouts at T1.

	Completers (at T1, n = 429) M (SD), or n (%)	Dropouts (at T1, n = 28) M (SD), or n (%)	T	χ^2^	p
HOP/control	293 (96%)/136 (90%)	13 (4%)/15 (10%)		5.68	0.02
Socio-demographic variables					
Age, years	42.5 (12.0)	41.0 (11.0)	−0.61		0.55
Female	284 (66%)	17 (61%)		0.93	0.63
Years of education	16.1 (3.9)	16.3 (4.4)	0.27		0.79
Born in Germany	396 (92%)	25 (89%)		0.39	0.47
Clinical variables					
Depressive symptoms (PHQ-9)	12.1 (5.9)	13.5 (5.5)	1.26		0.21
Number of psych. inpatient treatments	3.9 (4.3)	5.0 (6.8)	0.91		0.37
Years since first psych. diagnosis	14.2 (10.2)	14.7 (12.9)	0.25		0.80
Depressive disorder	309 (72%)	17 (61%)		1.65	0.20
Anxiety disorder	146 (34%)	13 (46%)		1.78	0.22
Psychosis/schizophrenia	88 (21%)	5 (18%)		0.11	1
Stigma-related variables					
Self-stigma	17.1 (7.5)	16.9 (7.7)	−0.13		0.90
Stigma stress	−0.1 (2.4)	−0.2 (2.8)	−0.31		0.76
Disclosure attitude (family/friends)	4.3 (1.3)	4.2 (1.4)	−0.59		0.56
Disclosure attitude (work/education)	3.5 (1.3)	3.5 (1.3)	−0.09		0.93
Shame^a^	4.0 (2.0)	4.1 (2.1)	0.29		0.78
Self-labeling^b^	4.6 (1.3)	4.7 (1.5)	0.56		0.58

a Rated from 1 (“I am not at all ashamed to have a mental illness”) to 7 (“I am very much ashamed to have a mental illness”).

b Rated from 1 (“I am perfectly mentally healthy”) to 7 (“I am severely mentally ill”).

### Intervention effects on primary and secondary outcomes

**Fig. 2:**
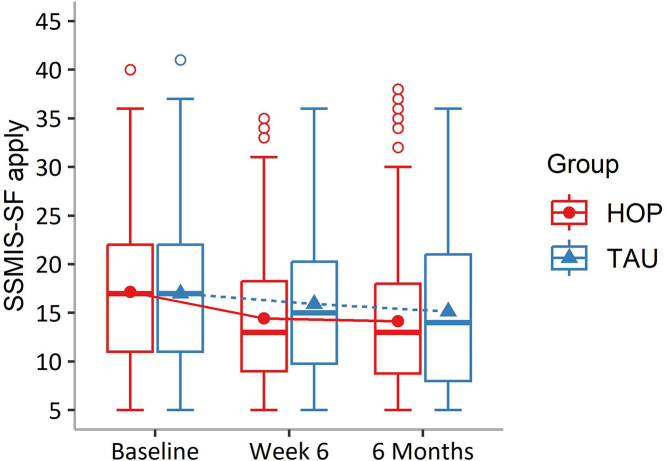
Box plot of self-stigma scores (SSMIS-SF, apply subscale) for HOP versus TAU, displaying the interquartile range with the median indicated by the horizontal line and means indicated by the circle/triangle.

Table 4 presents the results of the MMRM analysis of primary and secondary outcomes. With regards to our primary hypothesis and compared to the control group, HOP participants reported significantly lower self-stigma at T1 (Fig. 2).

**Table 4:** Mixed model for repeated measures (MMRM).

(−): decrease indicates improvement; (+): increase indicates improvement	Estimated group differences: M (95%-CI)		T1 (post)			T2 (follow-up)		
	Post (T1)	Follow-up (T2)	T	d	p	T	d	p
Self-stigma^a^ (−)	**−1.83 (−2.96 to −0.70)**	−1.20 (−2.36 to −0.05)	**−3.19**	**−0.24**	**0.0015**	−2.04	−0.16	0.042
Stigma stress^b^ (−)	−1.26 (−1.64 to −0.87)	−1.00 (−1.40 to −0.61)	−6.40	−0.52	<0.0001	−5.00	−0.41	<0.0001
(perceived stigma-related harm^b^) (−)	−0.53 (−0.79 to −0.26)	−0.46 (−0.73 to −0.20)	−3.94	−0.33	<0.0001	−3.39	−0.29	0.0007
(perceived coping resources^b^) (+)	0.74 (0.52 to 0.96)	0.55 (0.32 to 0.78)	6.50	0.57	<0.0001	4.74	0.42	<0.0001
Depressive symptoms^c^ (−)	−1.59 (−2.39 to −0.79)	−0.88 (−1.70 to −0.05)	−3.90	−0.28	<0.0001	−2.09	−0.15	0.037
Recovery^d^ (+)	1.73 (1.08 to 2.38)	0.81 (0.14 to 1.48)	5.20	0.41	<0.0001	2.39	0.19	0.017
Quality of life^e^ (+)	1.21 (0.42 to 1.99)	0.73 (−0.08 to 1.54)	3.01	0.20	0.0027	1.78	0.12	0.076
Intention to seek professional help^f^ (+)	0.20 (0.00 to 0.40)	0.05 (−0.16 to 0.25)	1.99	0.16	0.047	0.40	0.03	0.69
Attitudes to disclosure (family/friends)^g^	0.12 (−0.11 to 0.34)	0.09 (−0.14 to 0.32)	1.02	0.09	0.31	0.74	0.07	0.46
Attitudes to disclosure (work/education)^g^	0.21 (−0.03 to 0.44)	0.17 (−0.07 to 0.42)	1.74	0.16	0.083	1.39	0.13	0.16
Social inclusion^h^ (+)	1.74 (0.71 to 2.77)	0.27 (−0.78 to 1.33)	3.31	0.25	0.0010	0.51	0.04	0.61
Social Outcomes^i^ (+)	0.08 (−0.08 to 0.24)	0.09 (−0.08 to 0.26)	0.96	0.06	0.34	1.08	0.07	0.28
Self-Labeling^j^ (−)	−0.12 (−0.33 to 0.09)	−0.06 (−0.27 to 0.16)	−1.09	−0.09	0.28	−0.54	−0.05	0.59
Shame^j^ (−)	−0.33 (−0.64 to −0.02)	−0.17 (−0.48 to 0.15)	−2.10	−0.16	0.036	−1.03	−0.08	0.30

Positive group differences indicate an increase of the respective outcome in the HOP group as compared to control, and vice versa for negative estimates. Primary endpoint in bold; for means and SD of available cases see Table in Appendix.

a Self-Stigma of Mental Illness Scale-Short Form, subscale apply.^13^

b Stigma Stress Scale and its two subscales (harm and resources).^15^

c Patient Health Questionnaire (PHQ-9).^19^

d Self-Identified Stage of Recovery Scale (SISR-B).^33^

e EUROHIS-QOL.^16^

f General Help-Seeking Questionnaire.^23^

g Attitudes to Disclosure Questionnaire.^24^

h Experiences of Social Inclusion Scale.^20^

i Footnote Table 2.

j Footnotes Table 3.

In terms of secondary outcomes, HOP had significant positive effects compared to TAU on depressive symptoms and recovery at T1 and T2 and on self-stigma at T2. Effects on stigma stress were significant at T1 and T2, mainly driven by an increase in perceived coping resources (Table 4 and Table in Appendix). Significant intervention effects at T1, but not at T2, were obtained for quality of life, help-seeking intentions, shame and social inclusion. There were no significant intervention effects on disclosure attitudes at T1 or T2 (data available for the work-education/family-friends domains: 407/427 at T0, 371/403 at T1, and 317/367 at T2, respectively, equaling a range of 69–89% of those randomised for the work-education and of 80–93% for family-friends domain). In a MMRM sensitivity analysis adjusted for the partially nested design, HOP effects on the primary endpoint (self-stigma at T1) remained significant with −1.78 (95%-CI −2.94 to −0.63, d = 0.24, p = 0.0026). Sensitivity analyses with ANCOVAs supported the results. Descriptive data for all outcomes at T0, T1 and T2 are reported in the Appendix.

### Longitudinal mediation analysis of intervention effects on quality of life

**Fig. 3:**
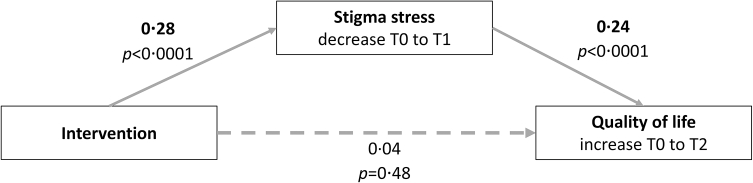
Longitudinal mediation model with difference scores for stigma stress (T0-T1; higher scores indicate higher reduction in stigma stress) and quality of life (T2-T0; higher scores indicate higher increase in quality of life). Structural equation modeling with standardised coefficients, N = 457, indirect (mediated) effect 0.07, p = 0.0010; total effect 0.10, p = 0.039.

The decrease in stigma stress from T0 to T1 fully mediated the relationship between the intervention and increase in quality of life from T0 to T2 (Fig. 3). Model fit could not be interpreted as the model was saturated (df = 0).

### Dose-response relationship

In an exploratory analysis, the relationship between the number of attended HOP sessions and change scores was significant for stigma stress at T1 ($r_{s}$ = 0.18, p = 0.0021), and T2 ($r_{s}$ = 0.15, p = 0.025), indicating higher reductions in stigma stress among those who attended more HOP sessions. Correlations with changes in self-stigma, depressive symptoms and quality of life were non-significant (all p-values > 0.25).

### Moderators of HOP outcomes

Based on our individual-participant data meta-analysis of six previous HOP RCTs,^34^ we tested age, gender, first-generation immigrant status, shame at baseline, as well as diagnoses (PTSD, schizophrenia, substance use) as potential moderators of HOP outcomes, in an exploratory analysis looking at data of HOP participants only and dummy-coding time of measurement. Younger age was associated with greater improvements of stigma stress (b = 0.04, p = 0.018) and depressive symptoms (b = 0.08, p = 0.030) among HOP participants from T0 to T2. More shame at baseline was related to stronger reductions in stigma stress at T1 (b = −0.35, p < 0.001) and T2 (b = −0.35, p < 0.001) compared to T0. The diagnoses of PTSD (p = 0.80), psychosis (p = 0.82) or substance use disorder (p = 0.57), respectively, did not affect HOP effects on self-stigma. All other effects were non-significant (p-values > 0.07).

### HOP and re-employment

Since disclosure decisions can affect re-employment,^35^ we examined possible HOP effects on re-employment among those 88 participants who were unemployed at baseline and for whom data on employment status was available at follow-up (T2). While two thirds of TAU participants remained unemployed at T2 (21 out of 32), this was the case for only half of the HOP participants (28 out of 56); the difference was not statistically significant (χ^2^ = 2.0, Fisher’s exact test two-sided p = 0.19, odds ratio 1.91, Cramer’s V 0.151, suggesting a small-to-medium effect^36^).

### Participants’ evaluations of HOP

At T1, all HOP participants responded to six items and judged the quality of the HOP programme. On average, HOP’s approach and content (M = 6.4, SD = 0.9) and the delivery of the group sessions (M = 6.3, SD = 0.9) were rated very positively (for both items from 1/very bad to 7/very good). HOP was seen as suitable and implementable under real-world conditions outside a research project (M = 5.8, SD = 1.2, from 1/not at all to 7/very well). Participants thought HOP had helped them with (non-)disclosure decisions and stigma coping (M = 5.7, SD = 1.3; from 1/not at all helpful to 7/very helpful) and would recommend HOP to others (M = 6.5, SD = 0.9; from 1/never to 7/certainly). The programme length of 4 × 2 h was considered about right (M = 3.6, SD = 1.4; with 1/too short, 4/exactly right, and 7/too long).

### Preliminary economic evaluation

Costs for delivering HOP were estimated and included time for peers to deliver HOP, costs for printing HOP materials and, in some sites, venue hire. Two peers facilitated each HOP group and received €100 per 2-h session, including travel and preparation time, resulting in €800 as compensation for both peers during the entire 4-session HOP course. Adding an average of €50 per course for printing costs and venue hire, this resulted in €850 per course. With six participants per HOP group, programme costs were estimated to be €142 per participant. The mean annual QALY difference between participants receiving HOP and those receiving TAU was estimated as 0.025. This resulted in an incremental cost-utility ratio point estimate of 5680 € per QALY gained. The cost-effectiveness acceptability curve (see Figure in the Appendix) revealed a probability of 75% at a MWTP threshold of 20,000 €, increasing to 78% at a MWTP threshold of 25,000 € and 81% at a MWTP threshold of 50,000 €.

## Discussion

### Hypotheses and HOP effectiveness

The findings of this multi-site pragmatic RCT support the real-world effectiveness of HOP for adults with different psychiatric diagnoses. Our first hypothesis was confirmed as HOP effectively reduced self-stigma, consistent with conceptual work^5^ and the findings of our previous meta-analysis of smaller HOP trials.^6^ Our second hypothesis was supported for several secondary outcomes which suggests that HOP improves clinical and social outcomes beyond the stigma domain when added to standard care, although change in depressive symptoms did not reach the conventional threshold for clinically significant change.^37^ The only exception were attitudes towards disclosure; this likely implies that HOP does not work by making participants more ‘pro disclosure’ but by empowering them to make informed personal choices in different settings, for or against disclosure.^38^

Our third hypothesis about the stability of HOP’s effects at follow-up was supported for some outcomes, including key stigma variables, depressive symptoms and, marginally, quality of life. This is noteworthy as HOP is a brief intervention, and so far there had been no evidence on HOP effects beyond two months. Our fourth hypothesis about HOP effects on re-employment, among those participants who were unemployed at baseline, was not confirmed, although effects were in the expected direction. This may be due to an underpowered analysis in a subsample of less than 20% of all participants. Future studies should re-examine this issue, including HOP effects on job-search self-efficacy and other employment-related outcomes, since attitudes towards disclosure affected re-employment in a previous longitudinal observational study.^35^

Our fifth hypothesis on HOP’s cost-effectiveness in terms of quality of life gains was supported, indicating a high probability that HOP would be accepted as cost-effective at a MWTP threshold range suggested for the German health-care system.^32^ During the maintenance phase after this trial, HOP groups cost about half as much as they did during the study, further improving HOP’s cost-effectiveness on a long-term basis.

### Pragmatic trial and outcome moderators

Recruitment took place in a broad range of contexts, from psychiatric inpatient wards over secondary outpatient mental health services to mutual support groups and recovery colleges, resulting in a socio-demographically and clinically very diverse sample of participants and strengthening the trial’s external validity. Only the recruitment from general practitioners failed, possibly because disclosure decisions may seem relatively less urgent in primary care.

Regarding moderators of intervention effects, younger age predicted better HOP effectiveness similar to a previous meta-analysis.^34^ Unlike in that meta-analysis, in the current trial more shame at baseline predicted better outcomes. This suggests that HOP, at least in this study, supported those most affected by shame and likely most in need of support. Consistent with HOP being a transdiagnostic programme, diagnoses did not affect outcomes.

### Implications

This study has some implications for future research. Studies should examine HOP’s effects on actual disclosure decisions and effects of disclosure decisions over time. As there are other interventions to reduce self-stigma, including narrative, psychoeducational and acceptance-based approaches,^1^ future work should compare their effectiveness and implementability as longer interventions, such as Narrative Enhancement and Cognitive Therapy,^39^ may be harder to implement. Future studies could examine active ingredients, e.g., compare HOP-online (which exists, but has to the best of our knowledge not been evaluated) versus HOP face-to-face, and compare long term effects of HOP with versus without additional booster sessions.

In terms of implications for practice, HOP should be offered to people with mental illness across different settings to reduce stigma’s impact. This recommendation is based not only on HOP’s effects on stigma-related variables, but on clinical and quality of life outcomes and the stability of its long-term effects for some of these outcomes. HOP is a flexible and cost-efficient programme, facilitating its use in real-world conditions. Finally, HOP was rated positively by participants which is encouraging for its future implementation and dissemination and in part explains the high demand in the current trial.

### Limitations

We cannot rule out contamination between trial arms. Research staff and participants were not blinded to group allocation. Psychiatric diagnoses and most outcomes were determined by self-report, and not all psychiatric symptoms were assessed. There might have been non-specific intervention effects, therefore future studies should use an active control. The internal consistency of the three-item help-seeking attitudes scale was low. The dose-response relationship may be confounded by participant motivation. At follow-up, some intervention effects were not significant and most effect sizes were small. Our economic analysis is limited as we did not have cost data beyond intervention delivery; future research should measure the impact of HOP on health service use and on educational and vocational outcomes. Finally, perceived public stigma or experienced discrimination should be measured in future studies as potential moderators of HOP outcomes.

### Conclusions

HOP as a peer-led group programme is an effective intervention to reduce stigma’s impact on adults with mental illness and to improve clinical and social outcomes. Due to its brevity and flexibility, it can be provided in various settings for people with different mental disorders.

## Contributors

CSch, GS, PWC, CR, NO and NR contributed to study design. CSch, CW, CK, RB, CSan, IP, NKG, WS, JW, NO, PB and AH contributed to conducting the study and to data collection. KR, CR and NR analysed the data, except for the cost-effectiveness analysis done by RK; KR, CR and NR have directly accessed and verified the underlying data reported in this manuscript. NR wrote the first draft of the manuscript, all authors contributed to the manuscript and approved its final version. All authors confirm that they had full access to all the data in the study and accept responsibility to submit for publication.

## Data sharing statement

After the publication of the Article, de-identified individual participant data will be made available for academic purposes. Data will be shared with researchers affiliated with academic institutions. Access will be granted upon request and a research proposal. Data sharing must comply with institutional ethics board approval and other relevant regulations. Requests will be reviewed by NR. If approved, a data-sharing agreement will be required.

## Declaration of interests

All authors declare that there is no conflict of interest regarding the content of this report.

## Appendix A Supplementary data

Appendix

EIIWS_StudyProtocol

Translated Abstract
