# Supplementary material for: Real-World Effectiveness of the Peer-Led Honest, Open, Proud Programme for Self-Stigma Among Adults With Mental Illness: A Pragmatic, Multicentre, Randomised Controlled Trial
Source: Lancet Reg Health Eur. 2026 Jun 19;67:101751. doi: 10.1016/j.lanepe.2026.101751 (PMC13310598; doi:10.1016/j.lanepe.2026.101751)

Appendix, Table. Descriptive statistics for HOP and control groups of available cases

(T0: n=306 HOP, n=151 control; T1: n=293 HOP, n=136 control; T2: n=272 HOP, n=129 control)

|  |  | Baseline/T0  M (SD) | Post/T1  M (SD) | Follow-up/T2  M (SD) |
| --- | --- | --- | --- | --- |
| Self-stigma ^a^ | HOP  Control | 17·16 (7·54)  16·98 (7·45) | 14·43 (6·77)  15·92 (7·27) | 14·12 (7·08)  15·13 (7·34) |
| Stigma stress ^b^ | HOP  Control | -0·05 (2·53)  -0·14 (2·33) | -1·49 (2·21)  -0·26 (2·31) | -1·55 (2·45)  -0·48 (2·27) |
| (perceived stigma-related harm ^b^ ) | HOP  Control | 4·26 (1·62)  4·14 (1·59) | 3·49 (1·55)  3·96 (1·62) | 3·49 (1·62)  3·94 (1·61) |
| (perceived coping resources ^b^ ) | HOP  Control | 4·32 (1·36)  4·28 (1·21) | 4·98 (1·19)  4·22 (1·42) | 5·04 (1·22)  4·42 (1·25) |
| Depressive symptoms ^c^ | HOP  Control | 12·56 (5·89)  11·44 (5·68) | 10·20 (5·26)  10·86 (5·25) | 10·25 (5·12)  10·09 (5·48) |
| Quality of life ^d^ | HOP  Control | 23·12 (5·92)  23·94 (6·01) | 24·85 (5·76)  24·27 (5·54) | 24·97 (5·92)  24·69 (5·86) |
| Recovery ^e^ | HOP  Control | 16·56 (4·37)  16·84 (4·06) | 17·89 (3·89)  16·30 (3·96) | 17·66 (4·01)  16·97 (3·62) |
| Intention to seek professional help ^f^ | HOP  Control | 5·55 (1·28)  5·60 (1·21) | 5·82 (1·16)  5·68 (1·21) | 5·79 (1·22)  5·77 (1·14) |
| Attitudes to disclosure  (family/friends) ^g^ | HOP  Control | 4·31 (1·33)  4·34 (1·29) | 4·50 (1·34)  4·41 (1·27) | 4·42 (1·34)  4·38 (1·24) |
| Attitudes to disclosure  (work/education) ^g^ | HOP  Control | 3·55 (1·35)  3·53 (1·33) | 3·72 (1·31)  3·47 (1·26) | 3·80 (1·30)  3·44 (1·27) |
| Social inclusion ^h^ | HOP  Control | 35·90 (7·03)  35·99 (7·17) | 37·58 (6·65)  35·79 (6·96) | 37·12 (7·27)  36·63 (6·67) |
| Social Outcomes ^i^ | HOP  Control | 4·09 (1·30)  4·13 (1·28) | 4·15 (1·25)  4·07 (1·32) | 4·13 (1·27)  3·98 (1·26) |
| Self-Labeling ^j^ | HOP  Control | 4·53 (1·30)  4·63 (1·28) | 4·44 (1·26)  4·60 (1·28) | 4·31 (1·29)  4·46 (1·27) |
| Shame ^j^ | HOP  Control | 3·89 (2·04)  4·11 (2·02) | 3·29 (1·84)  3·74 (1·96) | 3·25 (1·87)  3·57 (1·92) |

^a^ Self-Stigma of Mental Illness Scale-Short Form, subscale apply^13^; ^b^ Stigma Stress Scale and its two subscales (harm and resources)^15^; ^c^ Patient Health Questionnaire (PHQ-9)^19^; ^d^ EUROHIS-QOL^16^; ^e^ Self-Identified Stage of Recovery Scale (SISR-B)^22^; ^f^ General Help-Seeking Questionnaire^24^; ^g^ Attitudes to Disclosure Questionnaire^25^; ^h^ Experiences of Social Inclusion Scale^20^; ^i^ footnote Table 2; ^j^ footnotes Table 3.

Appendix, Figure. Cost-effectiveness acceptability curve for HOP compared to TAU


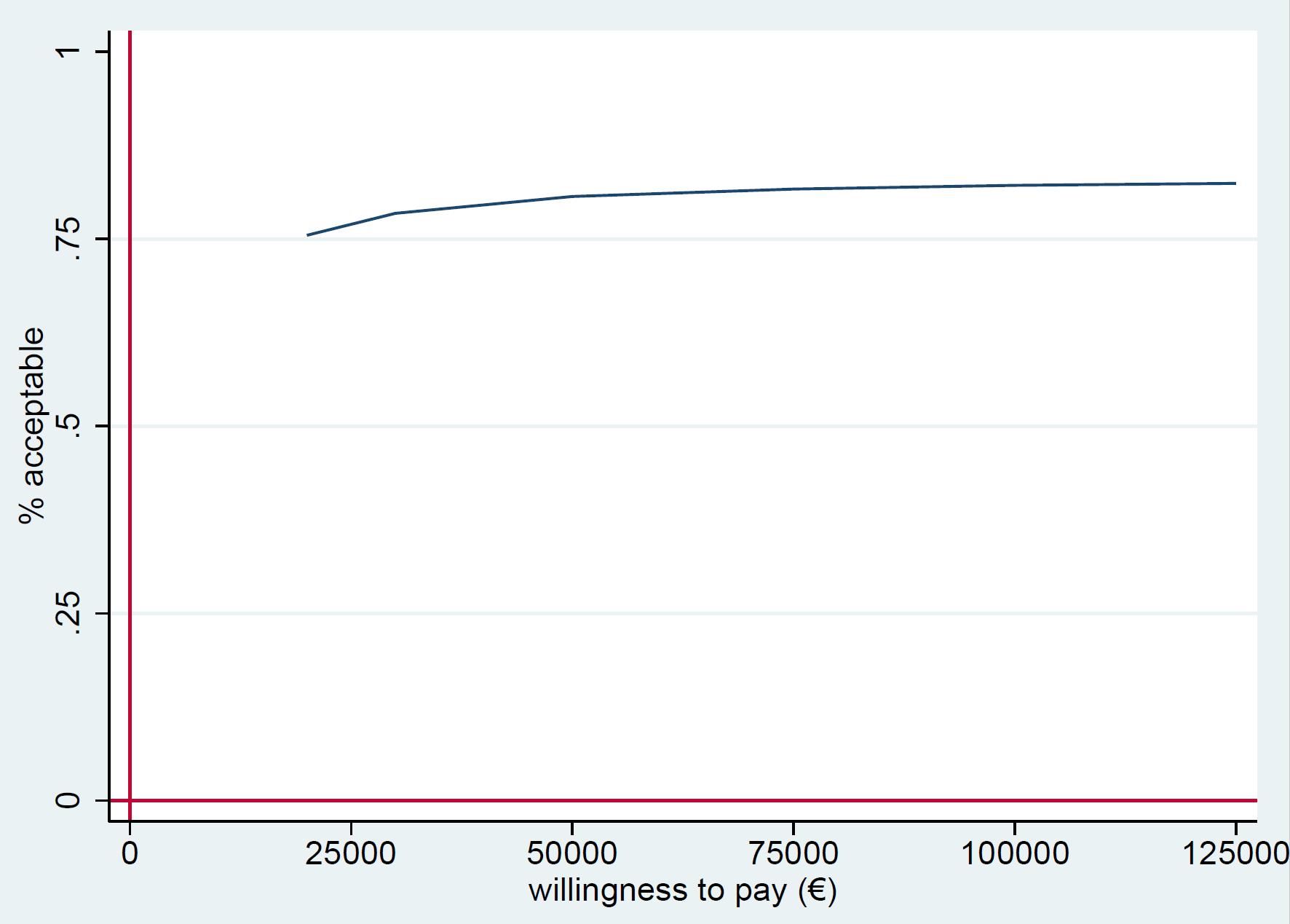

Supplement: Appendix [file mmc1.docx]
