# Supplementary material for: Real-World Effectiveness of the Peer-Led Honest, Open, Proud Programme for Self-Stigma Among Adults With Mental Illness: A Pragmatic, Multicentre, Randomised Controlled Trial
Source: Lancet Reg Health Eur. 2026 Jun 19;67:101751. doi: 10.1016/j.lanepe.2026.101751 (PMC13310598; doi:10.1016/j.lanepe.2026.101751)
Supplement: EIIWS_StudyProtocol [file mmc2.pdf]

Study Protocol

Effectiveness and Implementation of the Peer-Led Group Program "Honest, Open, Proud" (HOP) (German: In Würde zu sich stehen/IWS) to Reduce Self-Stigma Among Adults with Mental Illness

Project Acronym: EI-IWS

Funding and project duration: German Federal Ministry of Health, July 2023 – December 2025

Background

Due to public stereotypes against people with mental illness ("People with mental illness are stupid") many people with lived experience of mental disorders internalize these stereotypes, resulting in self-stigma ("Because I am mentally ill, I must be stupid"), shame, secrecy about the illness, and demoralization [1,2]. Self-stigma has serious consequences including giving up life goals, reduced help-seeking, social isolation, poorer clinical outcomes, increased risk for severe mental illness, and suicidality [3]. Stigma mainly is not diagnosis-specific but results from internalizing stereotypes about "the mentally ill." It is prevalent across severe mental disorders (e.g., schizophrenia, bipolar disorder [4,5]) as well as common mental disorders such as depression or anxiety disorders [6,7]. A transdiagnostic approach to reducing self-stigma is therefore appropriate.

Despite public attention around celebrities disclosing their mental illness, there is no evidence that self-stigma has declined. Our group's previous work shows that self-stigma and the burden of deciding whether to disclose one's illness as well as central barriers to coping with public and self-stigma remain [8]. Widely implemented interventions to reduce self-stigma suitable for daily practice are lacking.

The peer-led group program HOP (German: IWS) addresses this gap. HOP supports individuals in deciding whether or not to disclose their mental illness and thus supports participants in their coping with stigma. Our meta-analysis based on five small HOP randomized trials demonstrated that HOP reduces self-stigma on the short term ([9], confirmed in [10]). However, its effectiveness and implementability under real-world conditions have not yet been evaluated in a large pragmatic RCT.

Recent meta-analyses confirm both the severe negative impact of self-stigma on people with mental illness, the healthcare system and society [2], as well as the lack of effective and implemented interventions [11]. Cognitive and psychoeducational self-stigma interventions lack convincing evidence of effectiveness [11]. HOP is innovative in that it supports participants specifically with disclosure decisions—central to coping with both public and self-stigma.

Specific research questions and hypotheses

Question 1: Does HOP effectively reduce self-stigma among adults with mental illness in a pragmatic real-world trial?

Hypothesis 1: Self-stigma at T1 (after program completion/6 weeks after baseline) will be significantly lower among HOP participants compared to control participants.

Question 2: Does HOP improve secondary clinical and social outcomes?
Hypothesis 2: Compared to control participants, HOP participants will have improved outcomes in depressive symptoms, stigma stress, social inclusion, quality of life, help-seeking intentions, attitudes towards disclosure, and employment.

Question 3: Is HOP effective more than one month after program completion?
Hypothesis 3: At T2 (six months after baseline), self-stigma and secondary outcomes will be significantly improved among HOP participants compared to controls.

Question 4: Is HOP cost-effective in terms of quality of life gains?
Hypothesis 4: HOP is cost-effective in terms of quality of life gains.

Question 5: What facilitates or impedes the implementation of HOP in different settings?
Hypothesis 5: Implementation strategies co-developed with stakeholders during the first six months will result in good reach, study participation, intervention fidelity, and sustainability across settings (inpatient, outpatient, primary care, peer-support settings).

Design and Methods

Type I Effectiveness–Implementation Hybrid Trial

This study uses a hybrid design in which the primary focus is the real-world effectiveness of HOP, and the secondary focus is acceptability, feasibility, and preliminary effectiveness of the implementation intervention.

This hybrid approach allows both main study questions to be addressed simultaneously [12]:
(1) Does HOP work under pragmatic real-world conditions across regions and settings?
(2) Which factors support or hinder implementation, and how can implementation be strengthened to ensure successful dissemination and sustainability?

Participants / Inclusion and Exclusion Criteria

Inclusion criteria (all must be met):
– Self-reported mental illness per ICD-10, as self-stigma is a transdiagnostic problem and diagnosis did not predict HOP outcomes in prior studies [9]
– Age 18–60 years (to assess potential employment outcomes)
– written informed consent

Exclusion criteria (none must apply):
– acute suicidality or acute psychotic symptoms
– current substance use that interferes with HOP participation
– Primary substance use disorder without other psychiatric comorbidity
– Intellectual disability (IQ < 70)
– Insufficient German language skills to participate in the group program

Recruitment
To maximize generalizability, participants will be recruited from:
(i) inpatient units, day clinics, and outpatient clinics of psychiatric or psychotherapeutic hospitals;
(ii) private psychiatric and psychotherapeutic practices;
(iii) general practitioner (GP) practices;
(iv) peer-support groups and counseling centers for people with mental illness
(v) print and digital media.
Staff in these settings (including GP practice assistants) will be informed how to inform potential participants and to distribute flyers/posters. Interested individuals will contact study staff, who will conduct consent procedures, data collection, and randomization. Participants receive compensation for assessments and travel reimbursement.

Study Sites
The study will run at nine sites: four sites in Baden-Württemberg (Stuttgart, Konstanz, Heidelberg, Ulm), four in Bavaria (Günzburg, Augsburg, Munich, Regensburg), and in Leipzig (Saxony).
At each location, the study is conducted in cooperation between EX-IN (a German peer support organization), professionals in the psychiatric-psychotherapeutic care system (psychiatric clinics; registered psychiatrists and psychotherapists), and family doctors (GPs).

Intervention (HOP + TAU) and Control (TAU only)
HOP is a compact program of 4 sessions (2 hours each) [9]. Sessions 1–3 take place in weeks 1, 2, and 3, and session 4 (booster) in week 6. Groups consist of approximately 4–8 participants and are led by two peers— trained adults with lived experience of mental illness. Peer group facilitators are recruited via mutual support networks (e.g., "EX-IN"). They receive two days of structured training and continuous supervision throughout the study.

Main content of HOP sessions:
– Lesson 1: weighing pros/cons of disclosure in different situations
– Lesson 2: levels of disclosure and identifying appropriate recipients
– Lesson 3: telling one's story
– Lesson 4: booster session to reflect and update disclosure decisions
HOP does *not* aim to encourage disclosure; rather, participants learn to make strategic, context-dependent decisions for or against disclosure in different settings.

Control condition: TAU only. Participants may continue any ongoing psychiatric-psychotherapeutic or GP treatment or peer support.

Recruitment of HOP group facilitators
The study collaborates closely with regional German EX-IN associations (Bavaria, Baden-Württemberg, Saxony). EX-IN members, trained as peer-support workers, are well suited to facilitate HOP groups and to support implementation through their community networks.

Randomization
Immediately after baseline assessment (T0), participants will be block-randomized 2:1 (HOP:control). This facilitates the beginning of new HOP groups and supports recruitment by offering a higher probability of receiving HOP. Randomization is performed by closed opaque envelopes, provided by the Institute of Biometry, University of Ulm. A block randomization with varying block sizes (3, 6 or 9) will be used.

Assessment Times
T0: Baseline before randomization
T1: 6 weeks after baseline, and after the HOP booster session for HOP participants
T2: 6 months after baseline

Effectiveness Outcomes
The primary endpoint is self-stigma at T1, assessed via the 5-item self-concurrence subscale of the Self-Stigma of Mental Illness Scale–Short Form [15].

Secondary outcomes include:
– Quality of life (EUROHIS-QOL) [16]
– Depressive symptoms (PHQ-9) [17]
– Stigma stress (Stigma Stress Scale, 8 items) [18]
– Shame [19]
– Attitudes toward professional help-seeking (General Help-Seeking Questionnaire, 3-item subscale) [20,21]
– Attitudes toward disclosure [22]
– Recovery (Self-Identified Stage of Recovery, Part B, 4 items) [23]
– Social inclusion (Experiences of Social Inclusion Scale, 10 items) [24]
– Employment status [25]

Among participants in the HOP condition, program satisfaction is assessed at T1.
For details on the instruments used, see [9].

Sample Size
A previous German-speaking adult HOP RCT yielded an effect size of d = 0.4 for self-stigma 6 weeks after baseline [13]. With power = 0.80, alpha = 0.05 (two-sided), and a 12% sample-size increase for 2:1 randomization, the required total sample is 224 participants (149 HOP, 75 control) [13].

Statistical Analysis
The primary endpoint (self-stigma at T1) will be analyzed via intention-to-treat using mixed models for repeated measures (MMRM), with each HOP group as a nesting variable and adjustment for site. The two-sided significance threshold is $p < .05$.

Health Economic Evaluation
A cost-utility analysis will be conducted [27]. QALYs will be calculated using the EQ-5D-5L [28], and the cost-effectiveness of HOP in terms of gains in quality-adjusted life years will be examined.

Implementation Strategies

Implementation strategies will be applied at all study sites. In a participatory approach, strategies will be developed at the beginning of the project through interviews with people with lived experience, clinicians, GPs, relatives, and stakeholders [29]. The strategies will target RE-AIM dimensions [30]:

– Reach: informing and motivating potential participants
– Effectiveness: as above
– Adoption: uptake in different settings
– Implementation: fidelity assessment, qualitative interviews with leaders and participants
– Maintenance: identifying ways to sustain HOP beyond the study period.

Implementation Outcomes

Outcomes will be assessed quantitatively and qualitatively [30,31]. Initial interviews inform a questionnaire assessing barriers and facilitators, completed by participants and stakeholders. Fidelity will be measured with established checklists [13,26,32]. Adherence and dropout will be documented. The proportion of contacted institutions (e.g., GP practices) entering cooperation will be recorded.

Gender Aspects

Reviews do not show consistent gender effects on self-stigma [2]. However, HOP effects on depression appeared gender-specific in a California study (improvements for women only) [32]. Self-stigma may also impede help-seeking more strongly in men [2]. Gender (female, male, diverse/other) will therefore be analyzed regarding effectiveness, implementation, and dropout. We will aim for gender balance among advisory board members, study staff, and HOP group facilitators.

Participation

HOP is a peer-led intervention for people with mental illness; participation of people with lived experience is therefore central. In this study, peers (people with lived experience of mental illness) will be involved throughout, including study design, choice of measures, training of HOP group facilitators, supervision, recruitment, interpretation of findings, publication and dissemination of our results. EX-IN (German organization of peer support workers) associations will be actively involved. National and international experts in implementation science, general practice, and social psychiatry will contribute. If HOP proves effective and implementable, a version for relatives—already translated but not yet evaluated in Germany—will also be available.

Ethical and Legal Considerations

Previous HOP studies did not report adverse events. All participants (HOP and control) receive €20 per assessment. Minimal psychological burden due to the group setting or assessments cannot be excluded; therefore, informed consent is required. Participation is voluntary and may be discontinued at any time without giving a reason and without any negative consequences for the individual. Data will be pseudonymized. Recruitment begins after ethics approvals and after the registration of the trial in a publicly available online registry.

Use and Sustainability of Results
If HOP proves effective and feasible in real-world settings, it can be used throughout Germany. Results will be published with open access and in accessible formats for service users and relatives. EX-IN structures will remain after the project to support sustainability. The HOP workbook is freely available. A train-the-trainer model promotes long-term dissemination. HOP is non-commercial, low-cost, requiring only room availability and possible peer compensation. Strong synergies exist with the growing peer-support, trialogue, and EX-IN movement in Germany, supported by the national project partner „Aktion Psychisch Kranke“ (“Campaign for the Mentally Ill”).

Risks to Study Implementation
Risk 1: Delays in peer group leader recruitment.
Solution: Train more peers than needed to allow substitution.
Risk 2: Recruitment delays for study participants.
Solution: Recruit across multiple settings; compensate regional delays by other sites; add new study sites.

Project Management

Responsibilities / Involved Personnel
Prof. Nicolas Rüsch — University of Ulm, Public Mental Health
Project lead/Principal Investigator; HOP group facilitator training & supervision; analysis; sustainability; health policy

Jun.-Prof. Dr. Nathalie Oexle — University of Ulm
Deputy project lead; co-investigator; supervision of doctoral researchers; analysis & implementation support

Heike Liebsch, Jürgen P. Pfaff, Klaus Nuißl — EX-IN Associations BW, BY, Saxony
Cooperation partners within EX-IN, peer HOP facilitators

Prof. Anne Barzel — General Medicine, University of Ulm
Co-investigator; recruitment & implementation among GPs

Prof. Georg Schomerus — Univ. Leipzig
Co-investigator; site-specific contribution

Supporting Partners
Prof. P.W. Corrigan — Illinois Institute of Technology; program developer; project advisor
Prof. M. Wensing — Univ. Heidelberg; implementation science supervision
Prof. B. Mayer — Univ. Ulm; randomization

Prof. R. Kilian — Univ. Ulm; health economic supervision
Dr. Ch. Ruckes — Interdisciplinary Center for Clinical Trials, Mainz, Germany, biometry
Aktion Psychisch Kranke e.V. — national partner for dissemination and sustainability

References

1. Rüsch N (2023). The stigma of mental illness: Strategies against social exclusion and discrimination. Elsevier, Oxford, UK.
2. Dubreucq J, Plasse J, Franck N. Self-stigma in serious mental illness: A systematic review of frequency, correlates and consequences. Schizophr Bull. 2021;47:1261–1287.
3. Corrigan PW, Larson JE, Rüsch N. Self-stigma and the “why try” effect: Impact on life goals and evidence-based practices. World Psychiatry. 2009;8:75–81.
4. Brohan E, Elgie R, Sartorius N, Thornicroft G. Self-stigma, empowerment and perceived discrimination among people with schizophrenia in 14 European countries: The GAMIAN-Europe study. Schizophr Res. 2010;122:232–238.
5. Brohan E, Gauci D, Sartorius N, Thornicroft G. Self-stigma, empowerment and perceived discrimination among people with bipolar disorder or depression in 13 European countries: The GAMIAN-Europe study. J Affect Disord. 2011;129:56–63.
6. Curcio C, Corboy D. Stigma and anxiety disorders: A systematic review. Stigma Health. 2020;5:125–137.
7. Rüsch N, Hölzer A, Hermann C, Schramm E, Jacob GA, Bohus M, Lieb K, Corrigan PW. Self-stigma in women with borderline personality disorder and women with social phobia. J Nerv Ment Dis. 2006;194:766–773.
8. Rüsch N, Malzer A, Oexle N, Waldmann T, Staiger T, Bahemann A, Wigand ME, Becker T, Corrigan PW. Disclosure and quality of life among unemployed individuals with mental health problems: A longitudinal study. J Nerv Ment Dis. 2019;207:137–139.
9. Rüsch N, Kösters M. Honest, Open, Proud to support disclosure decisions and to decrease stigma’s impact among people with mental illness: Conceptual review and meta-analysis of program efficacy. Soc Psychiatry Psychiatr Epidemiol. 2021;56:1513–1526.
10. Sun J, Yin X, Li C, Liu W, Sun H. Stigma and peer-led interventions: A systematic review and meta-analysis. Front Psychiatry. 2022;13:915617.
11. Alonso M, Guillén AI, Muñoz M. Interventions to reduce internalized stigma in individuals with mental illness: A systematic review. Span J Psychol. 2019;22:e27.
12. Landes SJ, McBain SA, Curran GM. An introduction to effectiveness-implementation hybrid designs. Psychiatry Res. 2019;280:112513.
13. Rüsch N, Abbruzzese E, Hagedorn E, Hartenhauer D, Kaufmann I, Curschellas J, Ventling S, Zuaboni G, Bridler R, Olschewski M, Kawohl W, Rössler W, Kleim B, Corrigan PW. Efficacy of Coming Out Proud to reduce stigma’s impact among people with mental illness: Pilot randomised controlled trial. Br J Psychiatry. 2014;204:391–397.
14. Cunningham JA, Kypri K, McCambridge J. Exploratory randomized controlled trial evaluating the impact of a waiting list control design. BMC Med Res Methodol. 2013;13:150.
15. Corrigan PW, Michaels PJ, Vega E, Gause M, Watson AC, Rüsch N. Self-Stigma of Mental Illness Scale–Short Form: Reliability and validity. Psychiatry Res. 2012;199:65–69.
16. Brähler E, Mühlan H, Albani C, Schmidt S. Teststatistische Prüfung und Normierung der deutschen Versionen des EUROHIS-QOL Lebensqualität-Index und des WHO-5 Wohlbefindens-Index. Diagnostica. 2007;53:83–96.
17. Löwe B, Kroenke K, Herzog W, Grafe K. Measuring depression outcome with a brief self-report instrument: Sensitivity to change of the Patient Health Questionnaire (PHQ-9). J Affect Disord. 2004;81:61–66.

18. Rüsch N, Corrigan PW, Wassel A, Michaels P, Olschewski M, Wilkniss S, Batia K. A stress-coping model of mental illness stigma: I. Predictors of cognitive stress appraisal. Schizophr Res. 2009;110:59–64.
19. Rüsch N, Corrigan PW, Heekeren K, Theodoridou A, Dvorsky D, Metzler S, Müller M, Walitza S, Rössler W. Well-being among persons at risk of psychosis: The role of self-labeling, shame and stigma stress. Psychiatr Serv. 2014;65:483–489.
20. Wilson CJ, Deane FP, Ciarocchi J, Rickwood D. Measuring help-seeking intentions: Properties of the General Help-Seeking Questionnaire. Can J Couns. 2005;39:15–28.
21. Waldmann T, Staiger T, Oexle N, Rüsch N. Mental health literacy and help-seeking among unemployed people with mental health problems. J Ment Health. 2020;29:270–276.
22. Mayer L, Corrigan PW, Eisheuer D, Oexle N, Rüsch N. Attitudes towards disclosing a mental illness: Impact on quality of life and recovery. Soc Psychiatry Psychiatr Epidemiol. 2022;57:363–374.
23. Andresen R, Caputi P, Oades LG. Do clinical outcome measures assess consumer-defined recovery? Psychiatry Res. 2010;177:309–317.
24. Leemann L, Martelin T, Koskinen S, Härkänen T, Isola A-M. Development and psychometric evaluation of the Experiences of Social Inclusion Scale. J Hum Develop Capab. 2021:1–25.
25. Priebe S, Watzke S, Hansson L, Burns T. Objective social outcomes index (SIX): A method to summarize objective indicators of social outcomes in mental health care. Acta Psychiatr Scand. 2008;118:57–63.
26. Mulfinger N, Müller S, Böge I, Sakar V, Corrigan PW, Evans-Lacko S, Nehf L, Djamali J, Samarelli A, Kempter M, Ruckes C, Libal G, Oexle N, Noterdaeme N, Rüsch N. Honest, Open, Proud for adolescents with mental illness: Pilot randomized controlled trial. J Child Psychol Psychiatry. 2018;59:684–691.
27. Salize HJ, Kilian R. Gesundheitsökonomie in der Psychiatrie: Konzepte, Methoden, Analysen. Stuttgart: Kohlhammer; 2010.
28. Grochtdreis T, Dams J, König H-H, Konnopka A. Health-related quality of life measured with the EQ-5D-5L: Estimation of normative index values based on a representative German population sample and value set. Eur J Health Econ. 2019;20:933–944.
29. Powell BJ, Waltz TJ, Chinman MJ, Damschroder LJ, Smith JL, Matthieu MM, Proctor EK, Kirchner JE. A refined compilation of implementation strategies: Results from the ERIC project. Implement Sci. 2015;10:21.
30. Glasgow RE, Harden SM, Gaglio B, Rabin B, Smith ML, Porter GC, Ory MG, Estabrooks PA. RE-AIM planning and evaluation framework: Adapting to new science and practice with a 20-year review. Front Public Health. 2019;7:64.
31. Holtrop JS, Rabin BA, Glasgow RE. Qualitative approaches to use of the RE-AIM framework: Rationale and methods. BMC Health Serv Res. 2018;18:177.
32. Corrigan PW, Larson JE, Michaels PJ, Buchholz BA, Del Rossi R, Javier M, Castro D, Gause M, Rüsch N. Diminishing the self-stigma of mental illness by Coming Out Proud. Psychiatry Res. 2015;229:148–154.
