# Supplementary material for: Real-World Effectiveness of the Peer-Led Honest, Open, Proud Programme for Self-Stigma Among Adults With Mental Illness: A Pragmatic, Multicentre, Randomised Controlled Trial
Source: Lancet Reg Health Eur. 2026 Jun 19;67:101751. doi: 10.1016/j.lanepe.2026.101751 (PMC13310598; doi:10.1016/j.lanepe.2026.101751)
Supplement: Translated Abstract [file mmc3.docx]

**In Würde zu sich stehen als ein peer-geleitetes Gruppenprogramm, um Selbststigma bei Erwachsenen mit psychischer Erkrankung abzubauen: Pragmatische, multizentrische, randomisiert-kontrollierte Studie unter Alltagsbedingungen**

Von Nicolas Rüsch & Kollegen, *The Lancet Regional Health – Europe*

This translation in German was submitted by the authors and we reproduce it as supplied. It has not been peer reviewed. Our editorial processes have only been applied to the original abstract in English, which should serve as reference for this manuscript.

**Zusammenfassung**

**Hintergrund:** Das Stigma psychischer Erkrankung ist ein entscheidendes Hindernis für Genesung (Recovery), Hilfesuche und soziale Inklusion von Menschen mit psychischen Erkrankungen. Da diese mit Stigma und Diskriminierung konfrontiert sind, kämpfen viele mit der Entscheidung, ob sie ihre Erkrankung anderen gegenüber offenlegen. ‚In Würde zu sich stehen‘ (IWS; englisch: ‚Honest, Open, Proud‘/HOP) ist ein peer-geleitetes Gruppenprogramm, das Teilnehmer bei ihren Offenlegungsentscheidungen unterstützt (Peer, d.h. Person mit eigener Erfahrung psychischer Erkrankung). Die vorliegende Studie untersuchte die Wirksamkeit von IWS unter Alltagsbedingungen, Selbststigma zu verringern und sekundäre klinische und soziale Variablen zu verbessern, sowie die Kosteneffektivität von IWS.

**Methoden:** An dieser nicht-verblindeten, pragmatischen, parallelen, 2:1-randomisierten Studie nahmen Erwachsene mit psychischen Erkrankungen in erwerbsfähigem Alter teil, die an neun Standorten in Deutschland aus klinischen und nicht-klinischen Einrichtungen rekrutiert wurden. Die Teilnehmer wurden randomisiert entweder zu IWS und ihrer üblichen Behandlung (sog. treatment as usual/TAU, 2/3) zugeteilt oder nur zu ihrer üblichen Behandlung (nur TAU, 1/3). Die Erfolgsmaße wurden durch Selbstbeurteilung zu Beginn (Baseline), nach IWS (sechs Wochen nach Beginn) und wieder sechs Monate nach Beginn (Follow-up) erhoben. Der primäre Endpunkt war Selbststigma nach sechs Wochen. Interventionseffekte wurden ausgewertet durch sog. intention-to-treat-Auswertung. Die Studie war vorab registriert worden im Deutschen Register Klinischer Studien (DRKS; <https://drks.de/search/en/trial/DRKS00033314>).

**Ergebnisse:** Von Februar bis November 2024 wurden 457 Teilnehmer (durchschnittlich 42 Jahre alt, 66% Frauen, 92% in Deutschland geboren) in die Studie aufgenommen; 306 wurden IWS und üblicher Behandlung (Interventionsgruppe) zugeteilt, 151 nur üblicher Behandlung (Kontrollgruppe). Im Vergleich zur Kontrollgruppe zeigten IWS-Teilnehmer signifikant weniger Selbststigma nach sechs Wochen (-1·83, 95%-CI -2·96 to -0·70, d=-0·24, p=0·0015) und nach sechs Monaten (-1·20, 95%-CI -2·36 to -0·05, d=0·16, p=0·042). In Hinsicht auf sekundäre Erfolgsmaße führte die IWS-Teilnahme zu signifikanten Verbesserungen in Stigmastress, depressiven Symptome, Einstellungen zu Hilfesuche, Lebensqualität, Recovery (Genesungsorientierung), Scham und sozialer Inklusion nach sechs Wochen. Ein Teil dieser positiven Effekte blieb signifikant sechs Monate nach Studienbeginn. In einer gesundheitsökonomischen Evaluation war IWS kosteneffektiv in Bezug auf Lebensqualitätsgewinne. Es gab unter den Teilnehmern keine projektbezogenen unerwünschten Ereignisse.

**Interpretation:** IWS verringert bei Erwachsenen effektiv Selbststigma unter Alltagsbedingungen und sollte zusätzlich zur üblichen Versorgung in klinischen und nicht-klinischen Einrichtungen angeboten werden.

**Projektförderung:** Bundesministerium für Gesundheit (2523FSB22A, 2523FSB22B).
